# Tissue specificity and differential effects on in vitro plant growth of single bacterial endophytes isolated from the roots, leaves and rhizospheric soil of *Echinacea purpurea*

**DOI:** 10.1186/s12870-019-1890-z

**Published:** 2019-06-28

**Authors:** Valentina Maggini, Alessio Mengoni, Eugenia Rosaria Gallo, Sauro Biffi, Renato Fani, Fabio Firenzuoli, Patrizia Bogani

**Affiliations:** 1 Department of Biology, Laboratory of Plant Genetics, University of Florence, Via Madonna del Piano 6, I-50019 Sesto Fiorentino (Florence), Italy; 2 Department of Experimental and Clinical Medicine, University of Florence, Florence, Italy; 3 Research and Innovation Center in Phytotherapy and Integrated Medicine - CERFIT Careggi University Hospital, Florence, Italy; 4 Botanical Garden, Casola Valsenio, Italy

**Keywords:** Plant-biotic interactions, *Echinacea purpurea*, Endophyte, In vitro model, Growth promotion, Tissue specificity

## Abstract

**Background:**

*Echinacea*-endophyte interaction might affect plant secondary metabolites content and influence bacterial colonization specificity and plant growth, but the underlying mechanisms need deepening. An in vitro model, in which *E. purpurea* axenic plants as host species and *E. angustifolia* and *Nicotiana tabacum* as non-host species inoculated with single endophytes isolated from stem/leaf, root and rhizospheric soil, were used to investigate bacterial colonization.

**Results:**

Colonization analysis showed that bacteria tended to reach tissues from which they were originally isolated (tissue-specificity) in host plants but not in non-host ones (species-specificity). Primary root elongation inhibition as well as the promotion of the growth of *E. purpurea* and *E. angustifolia* plants were observed and related to endophyte-produced indole-3-Acetic Acid. Bacteria-secreted substances affected plant physiology probably interacting with plant regulators. Plant metabolites played an important role in controlling the endophyte growth.

**Conclusions:**

The proposed in vitro infection model could be, generally used to identify novel bioactive compounds and/or to select specific endophytes contributing to the host metabolism properties.

**Electronic supplementary material:**

The online version of this article (10.1186/s12870-019-1890-z) contains supplementary material, which is available to authorized users.

## Background

Plant microbe interplay is regulated by a plethora of signaling factors of different molecular nature (reviewed in [1, 2]). Plants can evolve with microbe communities by the perception of microbe secreted effector proteins that manipulate plant responses establishing pathogenic or beneficial symbiotic interactions [3–5].

Moreover, plant-associated endophytic bacteria colonize plants without apparently eliciting defense responses or injuring the plant. In some cases, endophytes induce a valuable promotion of plant growth [6–9], confer resistance to environmental stresses [10, 11] or contribute to ameliorate plant physical-chemical properties [12–16].

Plant-bacteria beneficial interactions are initiated by the chemotaxis of motile soil bacteria colonizing plant root surfaces [17]. Positive chemotaxis of *Rhizobium* spp. and other bacteria has been reported towards root and seed exudates, rich in various amino acids, sugars and phenolics, from legumes and other plants [18].

Then, endophytic colonization needs to overcome plant defense responses [19] and adapts itself to plant metabolism [20]. However, little is known about bacterial metabolic adaptation to the plant environment. Even though several genomics and proteomics studies have identified genes and proteins differentially regulated in the presence of plant root exudates, working as a switch to the endophytic remodeling [21–25], the identification of precise candidate molecules regulating plant- endophyte interaction appears extremely difficult.

A very important issue in the analysis of the host-endophyte relationships is the specificity of the interaction. In fact, despite of the capability of many microorganisms to invade any host, in literature there is a lot of information about the interaction between fungi [26–29] or bacteria [30–33] and their specific hosts.

In our previous paper concerning the works on the analysis of the microbiome of *Echinacea* species we have observed that different plant species and compartments select different endophytic bacterial strains [34]. Differences of bacterial communities among species and compartments could be due to the presence of differential bioactive compounds [35, 36], as alkamides, caffeic acid derivatives, polysaccharides and alkenes of which *Echinacea* species are rich [37]. Moreover, in the system *E. purpurea*-bacterial endophytes of the stem/leaf compartment, the plant-endophyte interaction affects the plant secondary metabolites content and it seems to drive the specificity of bacteria colonization in this important medicinal plant [12].

In this work, we have used plant tissue culture techniques in order to deepen the different aspects of the interaction between the bacterial endophytes isolated from *E. purpurea* stem/leaf, root and rhizospheric soil and *E. purpurea* plants as host species and *E. angustifolia* and *Nicotiana tabacum* as non-host species. The plant compartment specificity of the bacterial colonization has been evaluated using an in vitro model system in which a series of axenic plants of different species are inoculated with single selected endophytes for a total number of 6 endophytes isolated from each plant compartment (2 strains per compartment, Additional file 1). The effect of co-culture conditions on the growth of plant cells has been investigated by means of primary root elongation inhibition and bacterial promoting growth tests. Moreover, a bacterial growth test in the presence of host and non-host plant tissues macerates has been carried out.

## Results

### Tissue specificity of the bacterial colonization during plant infection

**Fig. 1:**
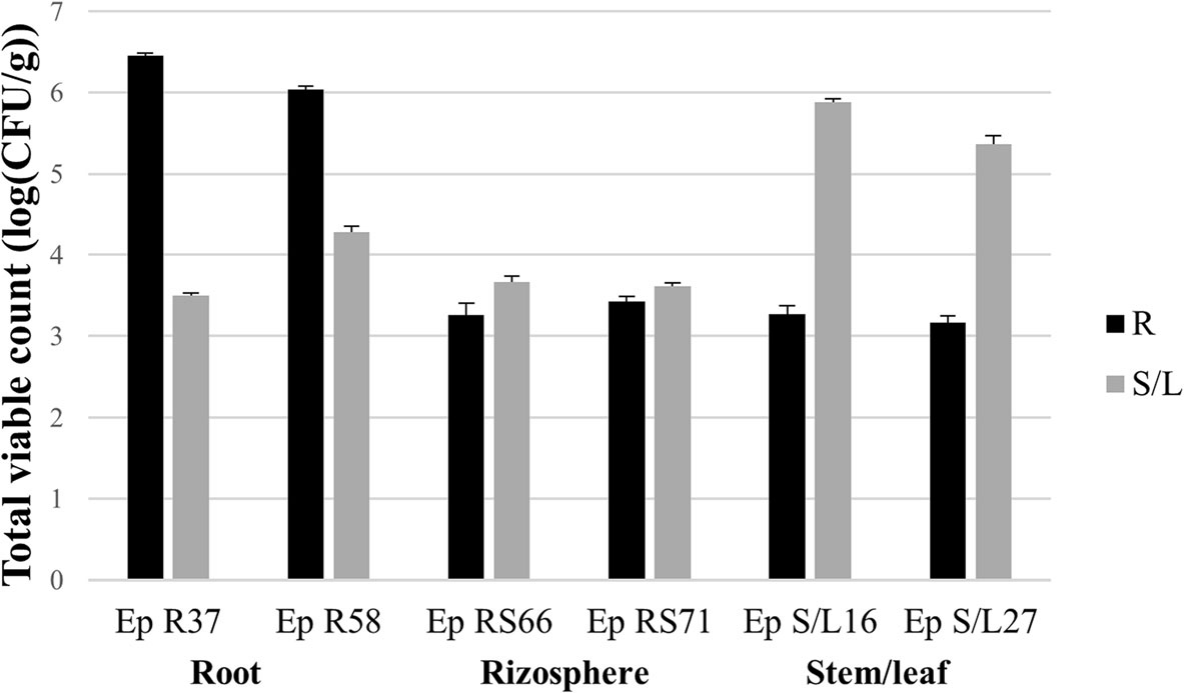
Total Viable Count (TVC) in *E. purpurea* root (R) and stem/leaf (S/L) tissues

Strains used in this work were selected from a culture collection previously established [34] according to their isolation in well-defined plant compartments. In particular, strains belonging to two genera, *Pseudomonas* and *Arthrobacter*, were selected, since strains of *Pseudomonas* were found with high frequency in the R compartment and strains of *Arthrobacter* were lacking in R, but highly representative of both RS and S/L compartments [34]. Additionally, *Pseudomonas* and *Arthrobacter* spp. were identified as PGP bacteria [38]. In order to fully exploit these qualities, within the panel of strains *Pseudomonas* and *Arthrobacter*, those with efficacious PGP properties [35] were selected (Additional file 1). Namely, six strains were chosen, *Pseudomonas* EpR37, R58, and *Arthrobacter* EpRS66, RS71, S/L16 and S/L27. These strains were used for in vitro *E. purpurea* plants infection experiments. Endophytic bacteria from each compartment were used to inoculate five axenic in vitro 2-months old *E. purpurea* plants; five plants, used as control, were inoculated with sterilized saline solution. In a preliminary infection experiment, thirty days after the infection, plants infected with S/L16 strain were analysed for bacterial colonization estimating the total viable count (TVC) as Colony Forming Units (CFU)/g into the host R and S/L tissues. Then, the infection experiment was repeated three times for each strain and the results recorded after 30 days. Data obtained revealed that the highest CFU/g was detected in the leaves (*p*_*anova*_ < 0.0001) when the plants were inoculated with strains from S/L compartment and in the roots (*p*_*anova*_ < 0.0001) when the infection was performed with R strains (Fig. 1 and Additional file 2). Finally, plants inoculated with RS strains showed less differences between compartments (the highest CFU/g was found in the leaves, *p*_*anova*_ < 0.05). The absence of bacteria in the control plant tissues and in the washing solutions confirmed the use of an axenic plant model and a successful sterilization procedure, respectively.

### Effects of bacterial infection on host plant growth

Data related to physiological parameters of control and infected *E. purpurea* plants were reported in Additional file 3. The analysis of the physiological parameters showed that the root-isolated strain *Pseudomonas* EpR37 had a borderline promoting effect (*p*_*t-test*_ *=* 0.05) on the plant fresh weight compared with the not-infected ones (Additional file 4a). Interestingly, the leaf/stem isolate *Arthrobacter* EpS/L16 induced a significant increase (*p*_*t-test*_ *=* 0.01) in the number of leaves (Additional file 4b). In the case of the infection of *E. purpurea* plants with the EpRS strains, no significant differences were observed (Additional file 4ab).

### IAA production

In order to check whether bacteria associated to *Echinacea* plants were able to produce IAA and there was a distinctive production among plant compartments, the six endophytic strains were tested for IAA production. The quantification of the produced IAA was estimated by a standard curve of IAA (Additional file 5). Data obtained revealed a gradient of IAA production EpS/L16 < EpRS71 < EpRS66 < EpR37 < EpR58 < EpS/L27 (Additional file 6).

### Inoculation of *E. purpurea* endophytic bacteria in non-host plants – colonization and effects on plant growth

To elucidate if the colonization of plant tissues and the effect on host plant growth was host-specific (i.e. related to the native host *E. purpurea*), endophytes were also used to inoculate non-host plants. The model plant *Nicotiana tabacum* and a non-host closely related species of *Echinacea* (*E. angustifolia*) were chosen. The experimental plan was the same used for the evaluation of *E. purpurea* inoculation.

### *N. tabacum* infection

Five *N. tabacum* plants were inoculated (i.e. infected) with each of the six utilized bacterial strains and five plants with saline solution (control). The experiment was carried out in triplicate for a total of 15 infected plants and 15 uninoculated control plants. Results indicated that two out of six strains only (EpR37 and EpRS66) were able to colonize plant tissues. Additionally, none of the strains was able to promote plant growth or leaf number, that is no overall influence on the plant physiology was detected (Additional file 7).

### Vertical agar plate assay

**Fig. 2:**
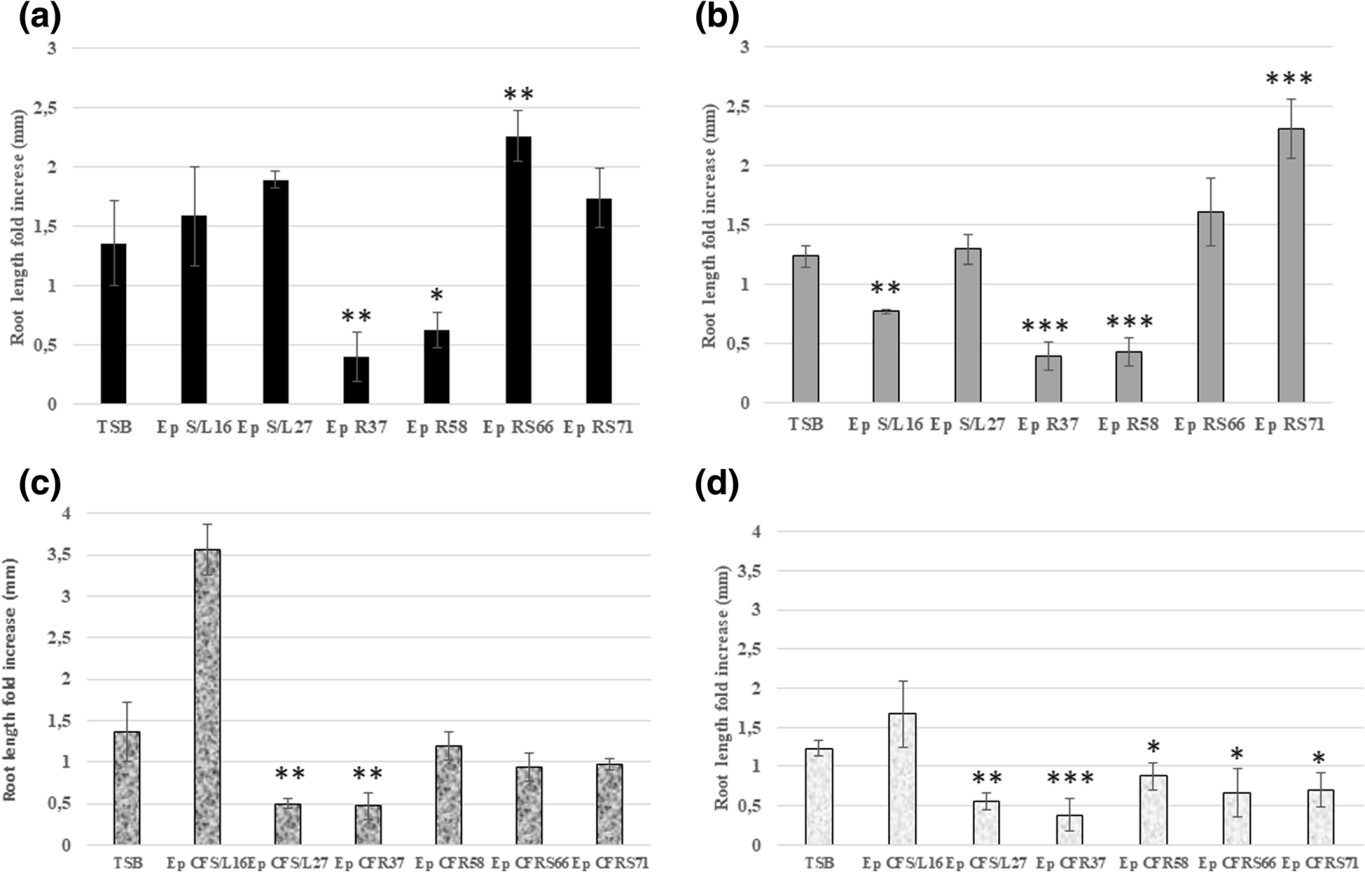
Primary root length elongation (mm) of seedlings of tobacco plants 7 days after inoculation with different *E. purpurea* (Ep) endophytes TSB: tryptic soy broth (negative control), CF: culture filtrate. Bars indicate standard errors between two replicates (*n* = 15). **a**, **c**: seedlings belonging to the > 2 cm class; (**b**), (**d**): seedlings belonging to the < 2 cm class. *p_*value*_ < 0.05; and ** *p*_*value*_ < 0.01; *** *p*_*value*_ < 0.001.

**Fig. 3:**
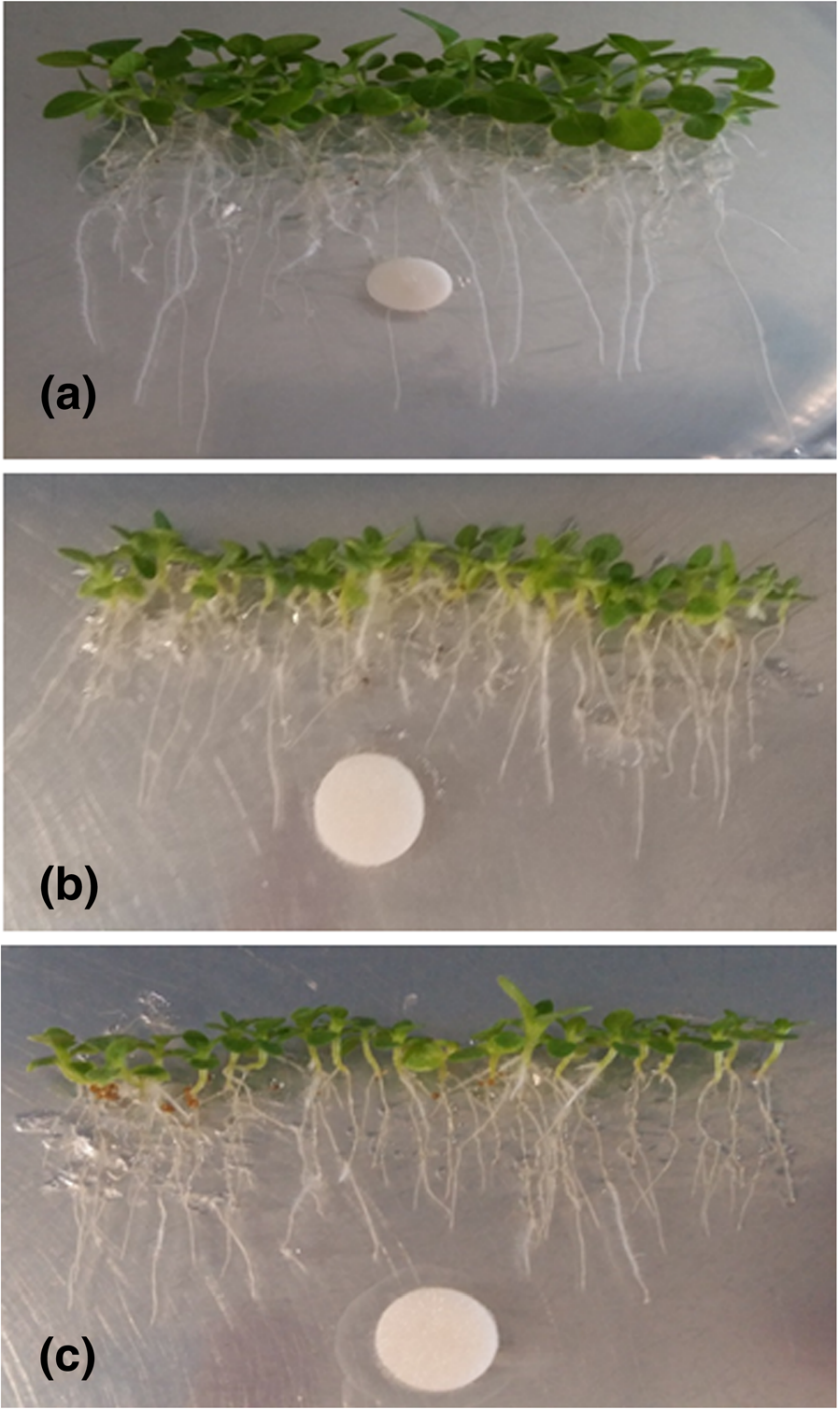
Modification of primary root morphology in vertically grown tobacco seedlings uninoculated (**a**) or inoculated with EpR58 (**b**) and EpR37 (**c**) root endophytes isolated from *E. purpurea*

Results in Figs. 2, 3 and Additional file 8 showed the effect of inoculation of tobacco plantlets, 15 days after germination with each endophyte. This effect related to the length of the primary root and to the changes in root apparatus due to the formation of branched roots and presence of root hairs in comparison with not-inoculated plantlets (Fig. 3). In particular, the length of the main root was in some cases either shorter or longer than control. More specifically, the inoculation of both R (EpR37 and EpR58) endophytic strains and their corresponding culture filtrates, induced a significant inhibition (*p*_*t-test*_ < 0.05 - < 0.001, respectively) of the primary root length either for seedlings grown at a distance more than 2 cm (> 2 cm; Fig. 2a, c) from the paper disc or those ones placed within 2 cm (< 2 cm; Fig. 2b, d). In contrast, seedlings inoculation with the rhizospheric strains EpRS66 (*p*_*t-test*_ < 0.01; Fig. 2a) and EpRS71 (*p*_*t-test*_ < 0.001; Fig. 2b) or with the culture filtrate of the stem/leaves strain EpS/L16 (*p*_*t-test*_ < 0.001; Fig. 2c) promoted a significant elongation of the primary root compared to the control. Concerning the culture filtrates inoculation, a general trend of root length inhibition was observed in comparison with control plants, especially in seedlings belonging to the < 2 cm class.

### *E. angustifolia* infection

Given the results on *E. purpurea* reported above, the infection of *E. angustifolia* species was performed only with the *Arthrobacter* EpS/L16. Five *E. angustifolia* plants were inoculated with EpS/L16 strain (infected) and five plants with saline solution (control) and the experiment was performed in triplicate. Data obtained revealed a contrasting colonization pattern for *E. angustifolia* plants in respect to *E. purpurea* ones. In fact, CFU/g was higher in the roots (1.81 × 10^7^ ± 4.85 × 10^5^; p_*anova*_ *<* 0.0001) than in the leaves (3.21 × 10^4^ ± 2.37 × 10^2^). As for *E. purpurea*, the inoculation of Ep S/L16 strain significantly influenced the number of leaves (*p*_*t test*_ *=* 0.03) (Additional file 9).

Overall, these data indicate that, at least for some of them, endophytic strains may show a tissue tropism related to the original tissue of isolation and that this tropism, and in part also the effect on plant physiology is host-specific. To shed light on the possible physiological interaction at cellular level an in vitro model system of tissue of *Echinacea* was developed and results are described in paragraphs below.

### Bacterial growth in different culture media

**Fig. 4:**
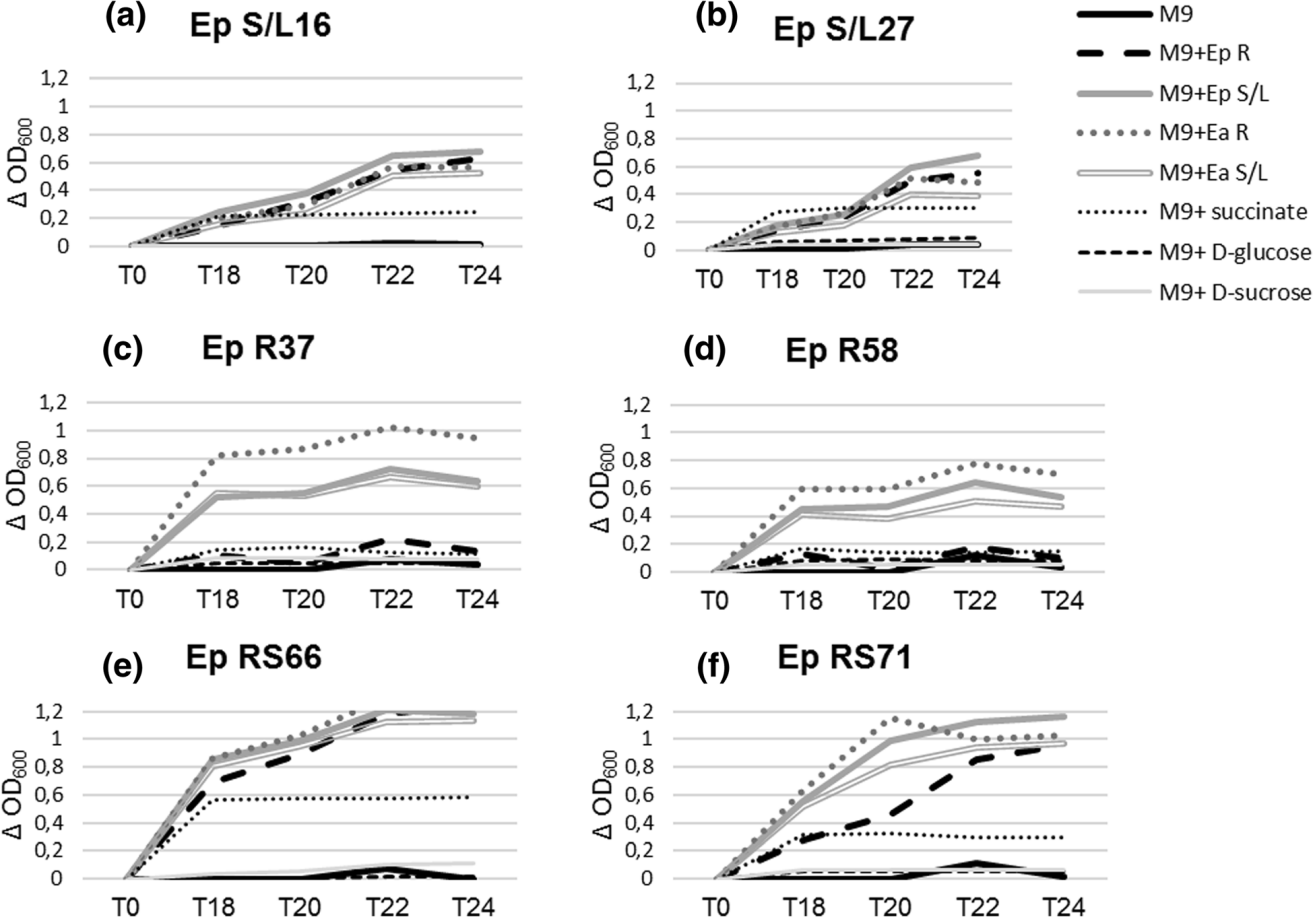
Growth of endophytes in M9 minimal medium supplemented with 1% D-glucose, 1% D-sucrose, 1% succinate and root (R) or stem/leaf (S/L) macerates of *E. purpurea* (Ep) and *E. angustifolia* (Ea). **a**: growth of Ep S/L16; **b**: growth of Ep S/L27; **c**: growth of Ep R37; **d**: growth of Ep R58; **e**: growth of Ep RS66; **f**: growth of Ep RS71

To investigate the possible metabolic basis of differential bacterial colonization, growth assays of endophytes with different carbon sources present in the medium (i.e. 1% D-glucose and 1% D-sucrose) or possibility present in root exudates (i.e. organic acids as 1% succinate) were performed. Additionally, growth assays with the whole plant tissue macerates were performed (Fig. 4). Most strains grew on succinate, but the cultures showed no increase in their OD values on D-glucose and D-sucrose. On the other hand, all strains grew well in root or stem/leaf macerates of *E. purpurea* and *E. angustifolia* with a final cell density higher than that of succinate-grown cells (*p*_*t-test*_ < 0.05) except for the R strains that grew less than the other strains in M9 supplemented with succinate and the macerate of *E. purpurea* roots.

## Discussion

In this work we investigated on the interaction between medicinal plants belonging to two different *Echinacea* species of the genera *Echinacea* (i.e. *E. purpurea* and *E. angustifolia*) and the endophytes isolated from in vivo *E. purpurea* tissues and rhizosphere [34]. To this purpose, an in vitro model of axenic plants inoculated with single endophytic strains (Ep S/L16, Ep S/L27, Ep RS66, Ep RS71, Ep R37 and Ep R58) was used [12]. Axenic plants were obtained sterilizing the seeds as previously described [12] and the absence of microbes was checked plating homogenized tissues (roots and stem/leaves) on bacterial nutrient medium and scoring bacteria growth after two, three and four days of incubation of the plates at 30 °C. We cannot a priori exclude the presence of residual Viable but Not Culturable Bacterial cells (as reported in [39]). However, after the infection experiment, the sterility check procedure by plating on was repeated for both control and inoculated plant tissues. Bacterial growth was observed only in inoculated plants, confirming the absence of viable and culturable endophytes in control plants.

Colonization analysis showed that the bacteria tended to reach their ecological origin niche in the host plant (e.g. Ep S/L strains were mainly found in *E. purpurea* leaves) but not in *E. angustifolia* confirming the tissue-specificity showed by the S/L endophytic pool in Maggini et al. (2017) and revealing the species-specificity of the investigated strains.

This hypothesis seems to be supported by the fact that alkamide biosynthesis in *E. purpurea* organs was influenced by the endophyte infection [12]. Moreover, *E. purpurea* and *E. angustifolia* endophytes from different plant compartments showed specific antibiotic resistance and production [36], suggesting that the bacterial communities could be structured by the communities themselves selecting bacterial phenotypes proper for plant colonization [35]. In fact, endophytes isolated from the leaves (EPS/L16 and 27) seemed specifically influence the number of leaves.

Notably, the six investigated strains were able to synthesize IAA at a different extent. Vertical agar plate assays showed the highest inhibition of the primary root elongation in vertical agar plate assays and the most severe morphological changes of tobacco seedlings roots as induced by the EpR37 and EpR58 endophytes and by the culture filtrate of the EpS/L27 strain, the highest IAA producers. These effects might be related to the IAA endophytic production since exogenous IAA was associated to root elongation and modification in different plant-microbe interaction systems [10].

Also, root and leaf macerates of both species were found to enhance the bacterial growth in comparison with minimal M9 medium. Moreover, the succinate promoted bacterial growth suggesting that the organic acids synthesized in plants might display an important role in controlling the endophyte growth, even though the most important factor for promoting bacterial growth remained to be determined. As known in literature, a simplified system for studying endophyte-host interactions is the establishment of dual culture in vitro protocols including endophytes and host plant tissues. Inhibitory or enhancer effects on the growth of endophytes or their corresponding hosts has been investigated [40–43] and in some cases it has been possible to select specific endophytes to ameliorate growth and productivity of plant hosts [44]. Thus, this approach might be a useful tool to get further insight into the identification of differential factors regulating the interaction between bacterial endophytes and *Echinacea* spp.

## Conclusions

The in vitro plant infection model used in this study could be generally used to deepen the physiology of the interaction in *Echinacea* and benefit from this to allow the identification of new bioactive compounds responsible of therapeutic properties of the plant. In the same time, this approach could allow to select specific endophytes contributing to the host metabolism properties.

## Methods

### Bacterial cultures

Six strains (Additional file 1) were selected from a collection previously described [34–36, 45] and set up from a pool of five *Echinacea purpurea* plants grown in a common garden at the “Il Giardino delle Erbe”, Casola Valsenio, Italy. The strains were separately collected from the roots (R) and stem/leaves (S/L) of the plants as well as from the rhizospheric soil (RS). Stock bacterial cultures (25% glycerol at − 80 °C) were grown at 30 °C on tryptone soy broth (TSB, Bio-Rad, USA) liquid medium or tryptone soy agar (TSA; Bio-Rad, USA) solid medium.

### Bacterial Indole-3-acetic acid (IAA) production

One colony for each strain was suspended in 3 ml of TSB liquid medium and the cultures were grown at 30 °C up to an OD_600_ = 0.5. Three ml of 1:10 dilution of a TSB solution, supplemented with 1 mg/ml L-tryptophan, were inoculated with 200 μl of each strain liquid culture as described previously [46]. After incubation over night at 30 °C, the absorbance (Abs) was measured at 600 nm. Then, 50 μl of Salkowsky reagent (50 ml, 35% perchloric acid and 1 ml 0.5 M FeCl3) were added to 50 μl of medium (single strain cultures). Absorbance (Abs Unit, AU) was measured after 30 min at 530 nm [46]. Active IAA production (Abs_530_/Abs_600_) was considered in relation to a standard curve (0.01–0.05-0.1-0.2-0.5-1.0-2.0-5.0 μM) of IAA (Sigma-Aldrich). Abs value for negative control (only medium) was also evaluated (0.08 AU). *E. coli* DH5α was used as internal control.

### In vitro plant material

*Echinacea purpurea* (L. Moench) and *Echinacea angustifolia* DC Hell seeds were gently provided by Dr. Sauro Biffi, Giardino delle Erbe. *Nicotiana tabacum* cv. Xanthi seeds were obtained from the Experimental Institute for Tobacco now renamed as Research Unit for Alternative Crops to Tobacco, CREA, Scafati (SA), Italy. Briefly, *Echinacea* and tobacco seeds were surface sterilized for 8 and 20 min, respectively, in 5% NaOCl solution, followed by three washes with sterile distilled water and then germinated and grown in Linsmaier & Skoog Medium (LS) including vitamins (Duchefa Biochemie, The Netherlands) at 24 ± 1 °C for a photoperiod of 16 h a day as previously respectively described [12, 47].

### Plant-bacteria interaction model

The analysis of the interaction among the selected strains and the *Echinacea* plantlets was carried out with the in vitro culture model developed by Maggini et al. [11]. Briefly, single bacterial *inocula* were incubated for two days at 30 °C and the bacterial suspensions adjusted to 8 × 10^8^ cfu/ml (OD_600_ = 1). The optical density (OD) was measured in a biophotometer (Eppendorf, Germany). Two months old *E. purpurea* plants were weighed (fresh weight in grams) and scored for the number of leaves. Then, five plants were transferred in Wavin flasks containing 50 ml LS basal medium and inoculated with 100 μl of a single bacterial suspension culture (we inoculated five plants for each bacterial strain). Five plants were used as control and were infected with 100 μl of sterilized 0.9% NaCl saline solution. Plants were then incubated in the growth chamber at 24 ± 1 °C. Thirty days after infection, plants from each experiment were scored again for both fresh weight and number of leaves. Biomass increase was reported as fresh weight fold increase (g) measured as (ffw-ifw)/ifw where ffw was the final weight of the whole plant after 30 days of culture after infection and ifw was the initial plant fresh weight. Then, both shoots and roots were separately collected, washed in saline solution (washing solution) and then sterilized in 1% (v/v) hypochlorite for 8 min. One gram of both fresh root and leave tissues were immediately used for the *in planta* endophyte bacterial growth analysis. The experiment was performed in triplicate.

### *In planta* bacterial growth analysis

In order to evaluate endophytes multiplication into host tissues, both roots and leaves samples from each experiment were separately homogenized in saline solution. One hundred microliters of the homogenate were serially diluted up to 10^− 7^/ml cells. Five replications of each dilution were plated on TSA medium. The washing solution and the distilled water after the last wash were also diluted to check the presence of bacterial cells on the surface of the tissues and the outcome of the sterilization procedure. Bacterial growth was scored after two days of incubation of the plates at 30 °C.

### Dual cultures methods to evaluate the effect of bacteria inoculation on plant root length inhibition: vertical agar plate assay

In order to evaluate the effect of bacterial inoculation on root growth, elongation experiments were performed as previously described [47]. Briefly, twenty *N. tabacum* seedlings of the same age and dimension were grown on 15 cm Petri dishes containing LS basal medium. One hundred μl of each 1 OD600 suspension cultures or 100 μl culture filtrates in TSB medium were inoculated on a sterilized filter paper disc placed 1 cm below the root tips of the seedlings, approximately at the center of the line of plants. Control treatments were made with 100 μl of TSB culture medium. Plates were incubated vertically in the growth chamber at 24 ± 1 °C and scored for root growth and morphology, after 7 days from treatments. Root growth was reported as root length fold increase (mm) measured as (fl-il)/il where fl was the length of primary root after 7 days of culture and il the initial length of primary roots. Each experiment was performed in duplicates.

### Bacterial promoting growth test

Endophytic strains were grown in TSB liquid medium up to an OD_600_ of 1.0 and the growth tests were performed by diluting bacterial cultures to an OD_600_ of 0.1 in microtiter plate with M9, M9 supplemented with 5 g l^− 1^ D-glucose or 5 g l^− 1^ D-sucrose or 10 g l^− 1^ succinate or 100 μl of root or stem/leaf macerates. The plate was placed in Infinite F200 PRO (TECAN, Salzburg, Austria) and incubated at 30 °C. The OD600 of the medium in the each well was recorded at every 2 h for 24 h. The change in OD600 was calculated with Microsoft Excel (Microsoft Corp., Redmond, WA, USA). If a well was dehydrated due to insufficient protection, the data from the well was excluded from the analysis. The procedure was performed in duplicate.

### Statistical analysis

The analysis of variance of the physiological parameters between infected and not infected *Echinacea* plants was carried out using One-way ANOVA (*p*_*value*_ < 0.05) or t-test. Mean separations were performed using the method of Tukey. The analyses were performed by using the modules present in the PAST program, version 3.15.

## Additional files

Additional file 1: Phenotypic features of the strains used in this work. Abbreviations: IAA, Indole-3-Acetic Acid; SPH, SideroPHore; EEA, Extracellular Enzymatic Activity; Ep, *Echinacea purpurea*; R, root; RS, rhizosphere; S/L, stem/leaves. (DOCX 21 kb)

Additional file 2: Comparison of bacterial colonization among *E. purpurea* (Ep) root (R) and stem/leaf (S/L) tissues after 30 days from the inoculation of endophytic strains. Total Vital Count (TVC) was computed in colony forming unit (CFU) / g of analysed tissue. Abbreviation: RS, rhizosphere. (DOCX 20 kb)

Additional file 3: Comparison of fresh weigh (∆FW) and number of leaves (∆NL) increases of *E. purpurea* (Ep) control and infected plants. ∆FW and ∆NL are reported as mean values (5 plants in triplicate) and calculated after 30 days. Abbreviations: R, root; RS, rhizosphere; S/L, stem/leaves; ns, not significant. (DOCX 21 kb)

Additional file 4: Effects on the growth of *E. purpurea* (Ep) in vitro plants of the infection with Ep root (R), rhizospheric (RS) and stem/leaves (S/L) endophytic strains. a) Fresh weigh (FW) and b) number of leaves (NL) increases of *E. purpurea* control (C) and infected (I) plants at the moment of the saline solution/bacterial inoculation (t0) and after 30 days (t30). FW and NL are reported as mean values (15 plants). The positive error bars were calculated on standard deviations of three experiments (*n* = 5 in each experiment). (DOCX 72 kb)

Additional file 5: Standard curve of indole-3-Acetic Acid (IAA). Active IAA production (Abs530/Abs600) was considered in relation to an IAA standard curve (serial dilution was 0.01–0.05-0.1-0.2-0.5-1.0-2.0-5.0 μM). (DOCX 46 kb)

Additional file 6: Active indole-3-Acetic Acid (IAA) production by endophytic strains used in this work. Abbreviations: Ep, *Echinacea purpurea*; R, root; RS, rhizosphere; S/L, stem/leaves. (DOCX 20 kb)

Additional file 7: Comparison of fresh weigh (∆FW) and number of leaves (∆NL) increases of *N. tabacum* control and infected plants. ∆FW and ∆NL are reported as mean values (5 plants in triplicate) and calculated after 30 days. Abbreviations: Ep, *Echinacea purpurea*; R, root; RS, rhizosphere; S/L, stem/leaves; ns, not significant. (DOCX 21 kb)

Additional file 8: Photographs showing the effect of inoculation of different *E. purpurea* (Ep) endophytes and their culture filtrates (CF) on primary root morphology and elongation in vertically grown tobacco seedlings uninoculated or inoculated. (a): TSB, tryptic soy broth (negative control); (b): Ep S/L27; (c): Ep CFS/L27; (d): Ep S/L16; (e): Ep CFS/L16; (f): Ep RS66; (g): Ep CFRS66; (h): Ep RS71; (i): Ep CFRS71; (l): Ep R58; (m): Ep CFR58; (n): Ep R37; (o): Ep CFR37. (DOCX 979 kb)

Additional file 9: Comparison of fresh weigh (∆FW) and number of leaves (∆NL) increases of *E. purpurea* and *E. angustifolia* control and infected with Ep S/L16 strain plants. ∆FW and ∆NL are reported as mean values (5 plants in triplicate) and calculated after 30 days. Abbreviation: ns, not significant. (DOCX 20 kb)

## Data Availability

All data generated or analyzed during this study are included in this published article [and its additional files] or are available from the corresponding author on reasonable request.
