# Supplementary material for: Tissue specificity and differential effects on in vitro plant growth of single bacterial endophytes isolated from the roots, leaves and rhizospheric soil of Echinacea purpurea
Source: BMC Plant Biol. 2019 Jun 28;19:284. doi: 10.1186/s12870-019-1890-z (PMC6598257; doi:10.1186/s12870-019-1890-z)
Supplement: Supplementary file 1 — Phenotypic features of the strains used in this work. Abbreviations: IAA, Indole-3-Acetic Acid; SPH, SideroPHore; EEA, Extracellular Enzymatic Activity; Ep, Echinacea purpurea; R, root; RS, rhizosphere; S/L, stem/leaves. (DOCX 21 kb) [file 12870_2019_1890_MOESM1_ESM.docx]

**Additional File 1**. Phenotypic features of the strains used in this work. Abbreviations: IAA, Indole-3-Acetic Acid; SPH, SideroPHore; EEA, Extracellular Enzymatic Activity; Ep, Echinacea purpurea; R, root; RS, rizosphere; S/L, stem/leaves; Tet, tetracycline; Rif, rifampicin; Clor, chloramphenicol; Str, streptomycin; Kan, kanamicin; Cipr, ciprofloxacin.

| **Strain** | **Genus level** | **GenBank** | **IAA** | **SPH** | **EEA** | | | | |
| --- | --- | --- | --- | --- | --- | --- | --- | --- | --- |
|  |  |  |  |  | **Protease** | **Phospholipase** | **Lipase TW80** | **Lipase Trib** | **Amylase** |
| Ep R37 | *Pseudomonas* sp. | KJ642522 | 0.97 | 1 | 0 | 0 | 0 | 0 | 0 |
| Ep R58 | *Pseudomonas* sp. | KJ642491 | 0.99 | 1 | 0 | 0 | 0 | 0 | 0 |
| Ep RS66 | *Arthrobacter* sp. | KJ642538 | 0.35 | 0 | 1 | 1 | 0 | 1 | 1 |
| Ep RS71 | *Arthrobacter* sp. | KJ642542 | 0.56 | 0 | 1 | 1 | 0 | 1 | 0 |
| Ep S/L16 | *Arthrobacter* sp. | KJ642432 | 0.56 | 0 | 1 | 1 | 0 | 1 | 1 |
| Ep S/L27 | *Arthrobacter* sp. | KJ642420 | 0.29 | 0 | 0 | 1 | 0 | 0 | 0 |

| **Strain** | **Genus level** | **Ab Res** | | | | | |
| --- | --- | --- | --- | --- | --- | --- | --- |
|  |  | **Tet** | **Rif** | **Clor** | **Str** | **Kan** | **Cipr** |
| Ep R37 | *Pseudomonas* sp. | 2.5 | 5 | 50 | 50 | 10 | 0 |
| Ep R58 | *Pseudomonas* sp. | 1.25 | 5 | 50 | 2.5 | 1 | 0 |
| Ep RS66 | *Arthrobacter* sp. | 0 | 0 | 5 | 1 | 10 | 0 |
| Ep RS71 | *Arthrobacter* sp. | 0 | 0 | 0 | 2.5 | 10 | 2.5 |
| Ep S/L16 | *Arthrobacter* sp. | 0 | 0 | 0 | 2.5 | 10 | 1 |
| Ep S/L27 | *Arthrobacter* sp. | 0 | 0 | 0 | 2.5 | 50 | 0.5 |
