# Supplementary material for: Tissue specificity and differential effects on in vitro plant growth of single bacterial endophytes isolated from the roots, leaves and rhizospheric soil of Echinacea purpurea
Source: BMC Plant Biol. 2019 Jun 28;19:284. doi: 10.1186/s12870-019-1890-z (PMC6598257; doi:10.1186/s12870-019-1890-z)
Supplement: Supplementary file 2 — Comparison of bacterial colonization among E. purpurea (Ep) root (R) and stem/leaf (S/L) tissues after 30 days from the inoculation of endophytic strains. Total Vital Count (TVC) was computed in colony forming unit (CFU) / g of analysed tissue. Abbreviation: RS, rhizosphere. (DOCX 20 kb) [file 12870_2019_1890_MOESM2_ESM.docx]

**Additional File 2**. Comparison of bacterial colonization among *E. purpurea* (Ep) root (R) and stem/leaf (S/L) tissues after 30 days from the inoculation of endophytic strains. Total Vital Count (TVC) was computed in colony forming unit (CFU) / g of analysed tissue. Abbreviation: RS, rizosphere.

| **Strain** | **Genus level** | **TVC** CFU/g | |  |
| --- | --- | --- | --- | --- |
|  |  | **Mean ± SD** | | *p_anova_ value* |
|  |  | **R** | **S/L** |  |
| **Ep R37** | *Pseudomonas* sp. | 2.86x10^6^ ± 1.70x10^5^ | 3.14x10^3^ ± 2.42x10^2^ | <0.001 |
| **Ep R58** | *Pseudomonas* sp. | 1.08x10^6^ ± 9.14x10^4^ | 1.91x10^4^ ± 2.89x10^3^ | <0.001 |
| **Ep RS66** | *Arthrobacter* sp. | 1.80x10^3^ ± 5.99x10^2^ | 4.60x10^3^ ± 8.94x10^2^ | <0.001 |
| **Ep RS71** | *Arthrobacter* sp. | 2.64x10^3^ ± 4.77x10^2^ | 4.06x10^3^ ± 4.39x10^2^ | <0.001 |
| **Ep S/L16** | *Arthrobacter* sp. | 1.86x10^3^ ± 3.91x10^2^ | 7.50x10^5^ ± 8.81x10^4^ | <0.001 |
| **Ep S/L27** | *Arthrobacter* sp. | 1.47x10^3^ ± 2.35x10^2^ | 2.35x10^5^ ± 5.59x10^4^ | <0.001 |
