# Supplementary material for: Tissue specificity and differential effects on in vitro plant growth of single bacterial endophytes isolated from the roots, leaves and rhizospheric soil of Echinacea purpurea
Source: BMC Plant Biol. 2019 Jun 28;19:284. doi: 10.1186/s12870-019-1890-z (PMC6598257; doi:10.1186/s12870-019-1890-z)
Supplement: Supplementary file 3 — Comparison of fresh weigh (∆FW) and number of leaves (∆NL) increases of E. purpurea (Ep) control and infected plants. ∆FW and ∆NL are reported as mean values (5 plants in triplicate) and calculated after 30 days. Abbreviations: R, root; RS, rhizosphere; S/L, stem/leaves; ns, not significant. (DOCX 21 kb) [file 12870_2019_1890_MOESM3_ESM.docx]

**Additional File 3**. Comparison of fresh weigh (∆FW) and number of leaves (∆NL) increases of *E. purpurea* (Ep) control and infected plants. ∆FW and ∆NL are reported as mean values (5 plants in triplicate) and calculated after 30 days. Abbreviations: R, root; RS, rizosphere; S/L, stem/leaves; ns, not significant.

| **Strain** | **Genus** | ***E. purpurea* plants** | | | | | |
| --- | --- | --- | --- | --- | --- | --- | --- |
|  |  | **∆FW** (mean ± SD) | |  | **∆NL** (mean ± SD) | |  |
|  |  | Control | Infected | *P _t-test_ value* | Control | Infected | *P_t-test_ value* |
| **Ep R37** | *Pseudomonas* sp. | 1.43 ± 0.35 | 2.30 ± 0.61 | 0.05 | 1.50 ± 1.32 | 0.55 ± 0.39 | ns |
| **Ep R58** | *Pseudomonas* sp. | 1.40 ± 0.40 | 1.72 ± 1.12 | ns | 1.50 ± 1.32 | 0.83 ± 0.28 | ns |
| **Ep RS66** | *Arthrobacter* sp. | 1.92 ± 0.79 | 1.98 ± 0.62 | ns | 1.07 ± 0.22 | 0.96 ± 0.27 | ns |
| **Ep RS71** | *Arthrobacter* sp. | 1.92 ± 0.79 | 1.81 ± 0.57 | ns | 1.07 ± 0.22 | 0.97 ± 0.29 | ns |
| **Ep S/L16** | *Arthrobacter* sp. | 0.71 ± 0.38 | 0.76 ± 0.24 | ns | 0.27 ± 0.20 | 0.94 ± 0.61 | 0.01 |
| **Ep S/L27** | *Arthrobacter* sp. | 0.85 ± 0.51 | 0.60 ± 0.47 | ns | 0.38 ± 0.23 | 0.48 ± 0.26 | ns |
