# Supplementary material for: Tissue specificity and differential effects on in vitro plant growth of single bacterial endophytes isolated from the roots, leaves and rhizospheric soil of Echinacea purpurea
Source: BMC Plant Biol. 2019 Jun 28;19:284. doi: 10.1186/s12870-019-1890-z (PMC6598257; doi:10.1186/s12870-019-1890-z)

**Additional File 4.** Effects on the growth of *E. purpurea* (Ep) *in vitro* plants of the infection with Ep root (R), rizospheric (RS) and stem/leaves (S/L) endophytic strains. a) Fresh weigh (FW) and b) number of leaves (NL) increases of *E. purpurea* control (C) and infected (I) plants at the moment of the saline solution/bacterial inoculation (t0) and after 30 days (t30). FW and NL are reported as mean values (15 plants). The positive error bars were calculated on standard deviations of three experiments (n=5 in each experiment).
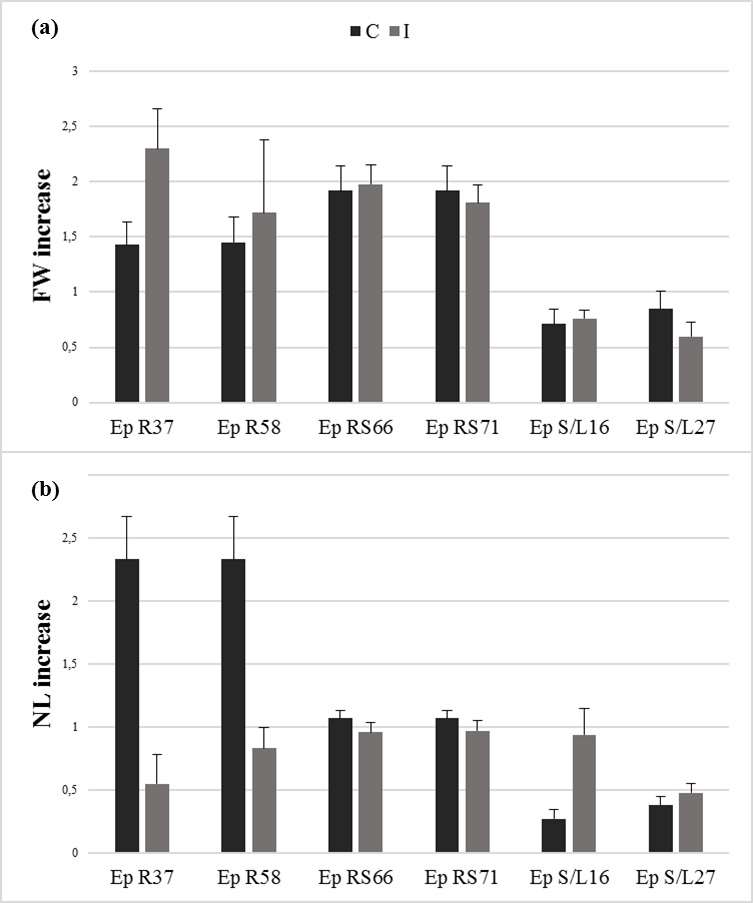

Supplement: Supplementary file 4 — Effects on the growth of E. purpurea (Ep) in vitro plants of the infection with Ep root (R), rhizospheric (RS) and stem/leaves (S/L) endophytic strains. a) Fresh weigh (FW) and b) number of leaves (NL) increases of E. purpurea control (C) and infected (I) plants at the moment of the saline solution/bacterial inoculation (t0) and after 30 days (t30). FW and NL are reported as mean values (15 plants). The positive error bars were calculated on standard deviations of three experiments (n = 5 in each experiment). (DOCX 72 kb) [file 12870_2019_1890_MOESM4_ESM.docx]
