# Supplementary material for: Tissue specificity and differential effects on in vitro plant growth of single bacterial endophytes isolated from the roots, leaves and rhizospheric soil of Echinacea purpurea
Source: BMC Plant Biol. 2019 Jun 28;19:284. doi: 10.1186/s12870-019-1890-z (PMC6598257; doi:10.1186/s12870-019-1890-z)

**Additional File 5.** Standard curve of indole-3-Acetic Acid (IAA). Active IAA production (Abs530/Abs600) was considered in relation to a IAA standard curve (serial dilution were 0.01-0.05-0.1-0.2-0.5-1.0-2.0-5.0 µM).


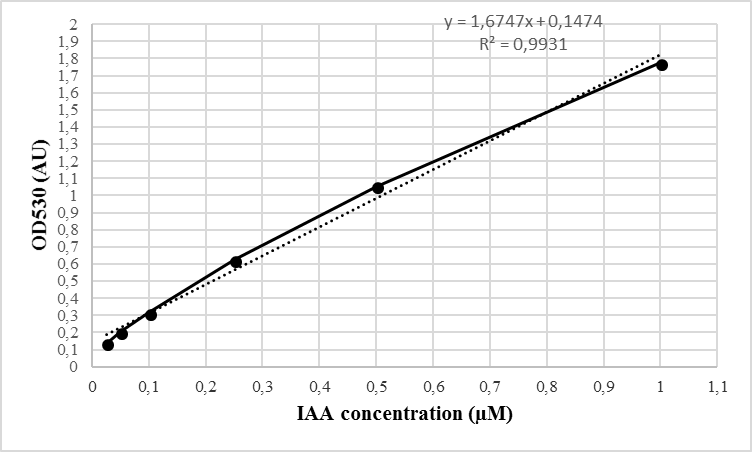

Supplement: Supplementary file 5 — Standard curve of indole-3-Acetic Acid (IAA). Active IAA production (Abs530/Abs600) was considered in relation to an IAA standard curve (serial dilution was 0.01–0.05-0.1-0.2-0.5-1.0-2.0-5.0 μM). (DOCX 46 kb) [file 12870_2019_1890_MOESM5_ESM.docx]
