# Supplementary material for: Tissue specificity and differential effects on in vitro plant growth of single bacterial endophytes isolated from the roots, leaves and rhizospheric soil of Echinacea purpurea
Source: BMC Plant Biol. 2019 Jun 28;19:284. doi: 10.1186/s12870-019-1890-z (PMC6598257; doi:10.1186/s12870-019-1890-z)
Supplement: Supplementary file 6 — Active indole-3-Acetic Acid (IAA) production by endophytic strains used in this work. Abbreviations: Ep, Echinacea purpurea; R, root; RS, rhizosphere; S/L, stem/leaves. (DOCX 20 kb) [file 12870_2019_1890_MOESM6_ESM.docx]

**Additional File 6.** Active indole-3-Acetic Acid (IAA) production by endophytic strains used in this work.

Abbreviations: Ep, Echinacea purpurea; R, root; RS, rizosphere; S/L, stem/leaves.

| **Strain** | **Genus level** | **IAA production µM** (mean±SD) |
| --- | --- | --- |
| *E. coli* DH5α | *Escherichia* sp. | 4.01 ± 0.22 |
| Ep R37 | *Pseudomonas* sp. | 0.52 ± 0.01 |
| Ep R58 | *Pseudomonas* sp. | 0.56 ± 0.02 |
| Ep RS66 | *Arthrobacter* sp. | 0.33 ± 0.01 |
| Ep RS71 | *Arthrobacter* sp. | 0.29 ± 0.01 |
| Ep S/L16 | *Arthrobacter* sp. | 0.25 ± 0.05 |
| Ep S/L27 | *Arthrobacter* sp. | 0.95 ± 0.01 |
