# Supplementary material for: Tissue specificity and differential effects on in vitro plant growth of single bacterial endophytes isolated from the roots, leaves and rhizospheric soil of Echinacea purpurea
Source: BMC Plant Biol. 2019 Jun 28;19:284. doi: 10.1186/s12870-019-1890-z (PMC6598257; doi:10.1186/s12870-019-1890-z)
Supplement: Supplementary file 7 — Comparison of fresh weigh (∆FW) and number of leaves (∆NL) increases of N. tabacum control and infected plants. ∆FW and ∆NL are reported as mean values (5 plants in triplicate) and calculated after 30 days. Abbreviations: Ep, Echinacea purpurea; R, root; RS, rhizosphere; S/L, stem/leaves; ns, not significant. (DOCX 21 kb) [file 12870_2019_1890_MOESM7_ESM.docx]

**Additional File 7.** Comparison of fresh weigh (∆FW) and number of leaves (∆NL) increases of *N. tabacum* control and infected plants. ∆FW and ∆NL are reported as mean values (5 plants in triplicate) and calculated after 30 days. Abbreviations: Ep, Echinacea purpurea; R, root; RS, rizosphere; S/L, stem/leaves; ns, not significant.

| **Strain** | **Genera** | ***N. tabacum* plants** | | | | | |
| --- | --- | --- | --- | --- | --- | --- | --- |
|  |  | **∆FW** (mean ± SD) | |  | **∆NL** (mean ± SD) | |  |
|  |  | Control | Infected | *P _t-test_ value* | Control | Infected | *P _t-test_ value* |
| **Ep R37** | *Pseudomonas* sp. | 2.40 ± 1.40 | 3.28 ± 0.94 | ns | 0.50 ± 0.32 | 0.85 ± 0.48 | ns |
| **Ep R58** | *Pseudomonas* sp. | 2.40 ± 1.40 | 2.72 ± 1.32 | ns | 0.50 ± 0.32 | 0.73 ± 0.48 | ns |
| **Ep RS66** | *Arthrobacter* sp. | 1.94 ± 2.15 | 3.79 ± 0.90 | ns | 0.45 ± 0.39 | 0.94 ± 0.30 | ns |
| **Ep RS71** | *Arthrobacter* sp. | 1.94 ± 2.15 | 0.33 ± 1.16 | ns | 0.45 ± 0.39 | 0.12 ± 0.39 | ns |
| **Ep S/L16** | *Arthrobacter* sp. | 9.02 ± 3.30 | 10.36 ± 4.75 | ns | 1.75 ± 0.57 | 1.64 ± 0.37 | ns |
| **Ep S/L27** | *Arthrobacter* sp. | 9.02 ± 3.30 | 7.40 ± 1.73 | ns | 1.75 ± 0.57 | 1.67 ± 0.87 | ns |
