# Supplementary material for: Tissue specificity and differential effects on in vitro plant growth of single bacterial endophytes isolated from the roots, leaves and rhizospheric soil of Echinacea purpurea
Source: BMC Plant Biol. 2019 Jun 28;19:284. doi: 10.1186/s12870-019-1890-z (PMC6598257; doi:10.1186/s12870-019-1890-z)

**Additional File 8.** Photographs showing the effect of inoculation of different *E. purpurea* (Ep) endophytes and their culture filtrates (CF) on primary root morphology and elongation in vertically grown tobacco seedlings uninoculated or inoculated. (a): TSB, tryptic soy broth (negative control); (b): Ep S/L27; (c): Ep CFS/L27; (d): Ep S/L16; (e): Ep CFS/L16; (f): Ep RS66; (g): Ep CFRS66; (h): Ep RS71; (i): Ep CFRS71; (l): Ep R58; (m): Ep CFR58; (n): Ep R37; (o): Ep CFR37.


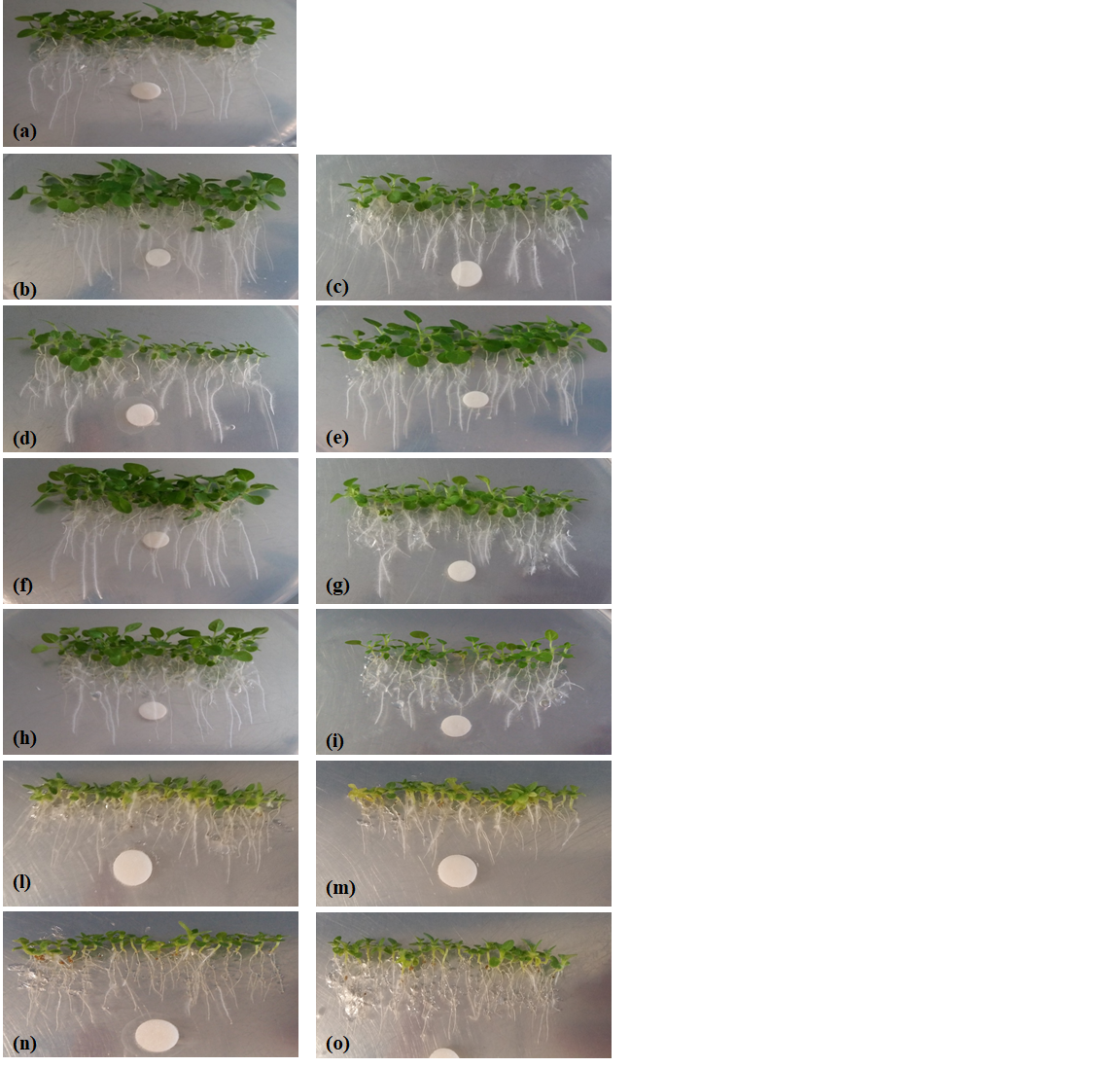

Supplement: Supplementary file 8 — Photographs showing the effect of inoculation of different E. purpurea (Ep) endophytes and their culture filtrates (CF) on primary root morphology and elongation in vertically grown tobacco seedlings uninoculated or inoculated. (a): TSB, tryptic soy broth (negative control); (b): Ep S/L27; (c): Ep CFS/L27; (d): Ep S/L16; (e): Ep CFS/L16; (f): Ep RS66; (g): Ep CFRS66; (h): Ep RS71; (i): Ep CFRS71; (l): Ep R58; (m): Ep CFR58; (n): Ep R37; (o): Ep CFR37. (DOCX 979 kb) [file 12870_2019_1890_MOESM8_ESM.docx]
