# Supplementary material for: Tissue specificity and differential effects on in vitro plant growth of single bacterial endophytes isolated from the roots, leaves and rhizospheric soil of Echinacea purpurea
Source: BMC Plant Biol. 2019 Jun 28;19:284. doi: 10.1186/s12870-019-1890-z (PMC6598257; doi:10.1186/s12870-019-1890-z)
Supplement: Supplementary file 9 — Comparison of fresh weigh (∆FW) and number of leaves (∆NL) increases of E. purpurea and E. angustifolia control and infected with Ep S/L16 strain plants. ∆FW and ∆NL are reported as mean values (5 plants in triplicate) and calculated after 30 days. Abbreviation: ns, not significant. (DOCX 20 kb) [file 12870_2019_1890_MOESM9_ESM.docx]

**Additional File 9.** Comparison of fresh weigh (∆FW) and number of leaves (∆NL) increases of *E. purpurea* and *E. angustifolia* control and infected with Ep S/L16 strain plants. ∆FW and ∆NL are reported as mean values (5 plants in triplicate) and calculated after 30 days. Abbreviation: ns, not significant.

| **Plants** | **∆FW** (mean ± SD) | | | **∆NL** (mean ± SD) | | |
| --- | --- | --- | --- | --- | --- | --- |
|  | Control | Infected | *P _t-test_ value* | Control | Infected | *p_t-test_ value* |
| ***E. purpurea*** | 0.71 ± 0.38 | 0.76 ± 0.24 | ns | 0.27 ± 0.21 | 0.94 ± 0.61 | 0.01 |
| ***E. angustifolia*** | 0.50 ± 0.28 | 0.52 ± 0.33 | ns | 0.43 ± 0.25 | 0.63 ± 0.23 | 0.03 |
